# The European Society of Paediatric Radiology launches European Diploma in Paediatric Radiology

**DOI:** 10.1007/s00247-018-4132-x

**Published:** 2018-05-17

**Authors:** Samuel Stafrace, Rutger A.J. Nievelstein, Maria Raissaki, On behalf of the European Society of Paediatric Radiology Education and Professional Development Committee

**Affiliations:** 1 Sidra Medical and Research Center, Doha, Qatar; 2 Department of Radiology & Nuclear Medicine, UMC Utrecht/Wilhelmina Children’s Hospital, P.O. Box 85500, 3508 GA Utrecht, The Netherlands; 3 Department of Radiology, Faculty of Medicine, University Hospital of Heraklion, University of Crete, Heraklion, Greece

**Keywords:** ᅟ

## Abstract

ᅟ

## Introduction

Paediatric radiology is a unique field of radiology defined by age as opposed to regional or systemic anatomy. The European Society of Paediatric Radiology (ESPR) was founded in 1963 and its mission was to bring together physicians involved in imaging children, contribute to the progress of paediatric imaging, and encourage training and education [1]. The ESPR Education and Professional Development Committee was set up in 2015 to (a) coordinate and update training and educational curricula, including the setup of a format for a European Diploma in Paediatric Radiology; (b) provide a communication channel relevant to education among national, international and subspecialty societies and the ESPR; (c) review the structure of general and subspecialty education in paediatric radiology; (d) receive reports from working groups on various aspects of education, on radiology trainee forum, review policies and procedures; (e) advise on and agree upon the society’s policy on continuing professional education, and (f) to liaise with (inter)national authorities including the European Society of Radiology, the European School of Radiology, the Radiology Section of the Union of European Medical Specialists and the European Accreditation Council for Continuing Medical Education. The year 2017 marks another milestone in the history of ESPR with regard to education and certification. Following several discussions and considerable time investment among the ESPR Education and Professional Development Committee members under the chairmanship of Dr. Rutger A.J. Nievelstein, the first cycle of the European Diploma in Paediatric Radiology has been launched.

## Vision

The ESPR decided to launch the diploma for a number of reasons. There is no set European reference standard for training in our subspecialty. Across many European countries, the training standard achieved is often assumed to be adequate, depending on the reputation of the institution where one undergoes their higher training. The level of such training can be very variable and reproducible evidence of the standard achieved is challenging. Paediatric radiology questions may constitute a minority in, or even be absent from, national radiology exams. The ESPR has a responsibility to ensure that this gap is filled. Acquisition of a standardised diploma confirms to the training specialists that they have followed a robust educational curriculum delivered by leaders in the field. Reassurance is also provided to their current or future employers through the knowledge that their employed paediatric radiologists are trained to a set standard and that such training has been accredited by the recognised specialist European body. The European Diploma in Paediatric Radiology is a measure of excellence, which certifies that the holder has a level of knowledge and competence in line with the requirements of the European Society of Radiology training curricula for paediatric radiology [2–4].

## Who should strive for this diploma?

The ESPR aims to target radiology residents, paediatric radiology fellows and junior faculty with an interest in paediatric radiology. Previous experience and training in paediatric radiology are practically mandatory for successful completion of the full cycle for the European Diploma in Paediatric Radiology. The ESPR Education and Professional Development Committee recommendation is that the diploma curriculum (educational programme) would be ideally covered during subspecialty training. Consequently, candidates should preferably be in at least their third year of national radiology training at the time of their first course and examination.

## Educational curriculum and format of the European Diploma in Paediatric Radiology

**Fig. 1:**
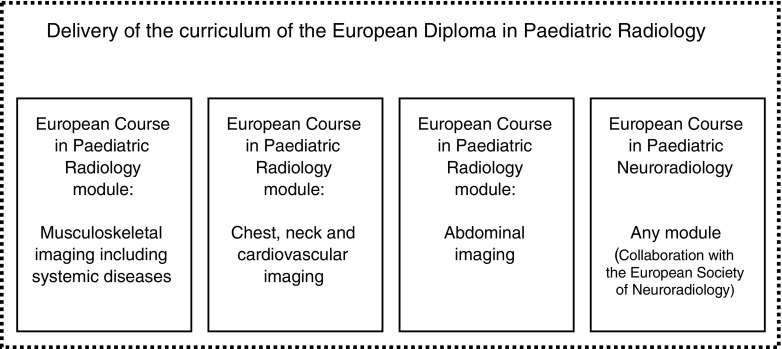
Courses required to complete the curriculum of the European Diploma in Paediatric Radiology

The European Course in Paediatric Radiology has been run successfully since 1992 through a rolling cycle of anatomically based topics (abdomen, chest including neck and cardiovascular, and musculoskeletal) [2]. As such, the model for the European Diploma in Paediatric Radiology will not be significantly different. The volume of competence and knowledge required to complete the diploma’s curriculum is based on the pediatric radiology sections of the European Society of Radiology training curriculum whose content had been provided by the ESPR under the leadership of Professor J.F. Chateil from Bordeaux, France [3]. After reappraisal by members of the ESPR Education and Professional Development Committee (Table 1), the current Level I, II and III European Society of Radiology training curricula for paediatric radiology [2, 3] were mapped into a format deliverable through the known European Course in Paediatric Radiology course layout. Based on this division, completion of the curriculum will follow the same simple model (Fig. 1). This will be done across the European Course in Paediatric Radiology curricula coupled with a further course covering paediatric neuroradiology components that will be delivered in partnership with the European Society of Neuroradiology. All courses will be followed by a relevant examination on their last day. An additional final assessment will take place during the ESPR annual meeting. The expected overall duration to earn the diploma is at least three years.

**Table 1:** Education and Professional Development Committee of the European Society of Paediatric Radiology

Curriculum section	Members responsible for reappraisal of each section
Cervicothoracic imaging (including neck and cardiovascular)	Dr. Anne Paterson, Dr. Eilish Twomey, Dr. Aurelio Secinaro
Abdominal imaging (including gastrointestinal and genitourinary)	Dr. Maria Raissaki, Dr. Samuel Stafrace
Musculoskeletal imaging (including multisystemic conditions)	Dr. Rutger A.J. Nievelstein, Dr. Thomas Augdal
Neuroradiology	In collaboration with the European Society of Neuroradiology (course organiser: Professor Maria Argyropoulou)

**Fig. 2:**
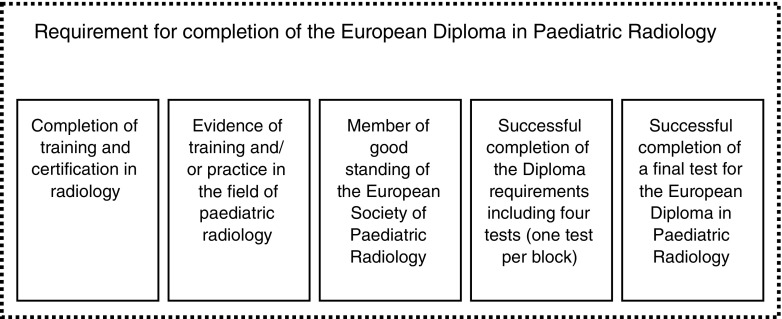
Overall requirements for completion of the European diploma in paediatric radiology

As the educational curriculum matures, more material will be delivered through online modules for ESPR members and European Course in Paediatric Radiology attendees. All steps required for completing the diploma are outlined in Fig. 2. In the end, the European Diploma in Paediatric Radiology will be granted to radiologists who have successfully completed the cycle of the European Course in Paediatric Radiology and the one European Course in Paediatric Neuroradiology and passed all the course examinations, followed by a final assessment during the ESPR annual meeting. The order of acquisition of the above steps is not important, as long as all steps are completed. A similar framework has been shown to be highly successful by the European Society of Neuroradiology [5].

The European Course in Paediatric Radiology will remain accessible to individuals who are not intending to complete the diploma and would like to simply enhance or renew their knowledge by attending the individual courses and targeting the body systems in question. The ESPR remains committed to keeping both the location and the cost of the three European Course in Paediatric Radiology modules as accessible as possible. Geography or cost should not act as barriers to candidates who wish to complete this training.

## Inaugural course

The first eligible European Course in Paediatric Radiology delivering a section of the newly mapped European Diploma in Paediatric Radiology curriculum was held in Utrecht, the Netherlands, on October 11–13, 2017. The course covered paediatric musculoskeletal imaging. An international specialist faculty kindly agreed to deliver this teaching material through didactic lectures and interactive sessions within the boundaries of the diploma curriculum. Sixty-one of the 150 course attendees participated in the examination at the end of the course.

The inaugural assessment of the European Diploma in Paediatric Radiology will be held in June 2020 during the ESPR annual meeting and after completion of the first eligible European Course in Paediatric Radiology three-part cycle. Final assessment for the diploma will then be available at every ESPR annual meeting. It will be the responsibility of the Education and Professional Development Committee of the ESPR to run this assessment.

## Conclusion

With the launch of the European Diploma in Paediatric Radiology, the ESPR is providing a new European standard of competence and certification. We urge senior European paediatric radiologists and paediatric radiology experts outside Europe to embrace this initiative by encouraging trainees and fellows to attend the European Course in Paediatric Radiology cycle and, if called upon by the ESPR, through their delivery of lectures and educational material in line with the diploma curriculum.
